# Improving adherence to antiretroviral treatment in Uganda with a low-resource facility-based intervention

**DOI:** 10.3402/gha.v7.24198

**Published:** 2014-06-04

**Authors:** Celestino Obua, Joshua Kayiwa, Paul Waako, Göran Tomson, Hudson Balidawa, John Chalker, Dennis Ross-Degnan, Rolf Wahlstrom

**Affiliations:** 1 Department of Pharmacology and Therapeutics, School of Biomedical Sciences, College of Health Sciences, Makerere University, Kampala, Uganda; 2 Data Department, Joint Clinical Research Centre, Kampala, Uganda; 3 Department of Public Health Sciences, Division of Global Health (IHCAR), Karolinska Institutet, Stockholm, Sweden; 4 Medical Management Centre (MMC), Karolinska Institutet, Stockholm, Sweden; 5 The AIDS Control Programme, Ministry of Health, Kampala, Uganda; 6 Centre for Pharmaceutical Management, Management Sciences for Health, Arlington, VA, USA; 7 Department of Population Medicine, Harvard Medical School, Boston, MA, USA

**Keywords:** antiretroviral therapy, adherence, management, staff motivation, intervention, Uganda

## Abstract

**Objective:**

To assess the effects of facility-based interventions using existing resources to improve overall patient attendance and adherence to antiretroviral therapy (ART) at ART-providing facilities in Uganda.

**Methods:**

This was an interventional study which tracked attendance and treatment adherence of two distinct cohorts: experienced patients who had been on treatment for at least 12 months prior to the intervention and patients newly initiated on ART before or during the intervention. The interventions included instituting appointment system, fast-tracking, and giving longer prescriptions to experienced stable patients. Mixed-effects models were used to examine intervention effects on the experienced patients, while Cox proportional hazards models were used to determine the intervention effects on time until newly treated patients experienced gaps in medication availability.

**Results:**

In all, 1481 patients’ files were selected for follow-up from six facilities – 720 into the experienced cohort, and 761 into the newly treated cohort. Among patients in the experienced cohort, the interventions were associated with a significant reduction from 24.4 to 20.3% of missed appointments (adjusted odds ratio (AOR): 0.67; 95% confidence interval (CI): 0.59–0.77); a significant decrease from 20.2 to 18.4% in the medication gaps of three or more days (AOR: 0.69; 95% CI: 0.60–0.79); and a significant increase from 4.3 to 9.3% in the proportion of patients receiving more than 30 days of dispensed medication (AOR: 2.35; 95% CI: 1.91–2.89). Among newly treated patients, the interventions were associated with significant reductions of 44% (adjusted hazard rate (AHR): 0.56, 95% CI: 0.42–0.74) and 38% (AHR: 0.62; 95% CI: 0.45–0.85) in the hazards of experiencing a medication gap of 7 and 14 days or more, respectively.

**Conclusions:**

Patients’ adherence was improved with low-cost and easily implemented interventions using existing health facilities’ resources. We recommend that such interventions be considered for scale-up at national levels as measures to improve clinic attendance and ART adherence among patients in Uganda and other low-resource settings in sub-Saharan Africa.

In resource-limited settings, antiretroviral therapy (ART) programs strive to maintain high numbers of patients taking the life-long medications as countries scale-up ART accessibility (1–5). Adherence to treatment is one of the most important predictors of survival for individuals with HIV and AIDS (6, 7). For most HIV regimens, patients need to achieve >95% level of adherence so as to avoid developing drug resistance (8). For 95% adherence, missing even one pill in a week on a regimen of two pills per day could lead to developing resistance to therapy.

Several factors such as distance to health facilities, availability and affordability of ARVs, lack of money for transport and food, quality of life during ARV, long waiting time, and congestion at health facilities influence adherence to ART (8, 9). Some of these factors require a programmatic approach, which may be difficult to address in a number of resource-limited settings (10, 11). Initiatives implemented at facility level, within facility resources, can affect a large number of patients more efficiently and are thus more sustainable. However, they need to be easy to introduce, implement, and should be affordable in terms of costs. In the past, initiatives designed to improve adherence have included training health personnel on ART supply chain management, HIV prevention, clinical care, counseling, community support programs, and use of experienced ART patients (expert clients) who provide support to other patients who are newly commencing treatment (6, 8). Frequently, such initiatives rely on substantial financial inputs that are often impossible to sustain by the health facilities in resource-limited settings (8, 12).

The current health-care system in Uganda was primarily designed for acute as opposed to chronic care. The system could be adapted to deliver chronic care through interventions targeted at improving clinic attendance and ART adherence. Many health facilities offering ART services lack systematic appointment systems, which results in over-crowding when patients come early hoping to be first to receive care. Subsequently, the congestion that develops negatively affects the quality of services as well as patient satisfaction with care (13). Furthermore, in many clinics, experienced patients already stabilized on ART are still required to present for care and go through the entire clinical process even if the only reason for their visit is to receive medication refills. To relieve congestion, these patients could be fast tracked to the pharmacy and/or dispensed longer durations of therapy.

In this study, we set out to determine the extent to which a set of easily implemented health facility-based interventions that used already existing resources at health facilities, mainly designed to reduce clinic congestion as well as patient waiting time, would improve clinic attendance and adherence among patients seeking ART services at Ugandan health facilities.

## Methods

### Study setting and design

Six district-level public hospitals previously determined to have poor adherence performance to ART were enrolled in this study based on the results of a previous national survey (8). All of these clinics had poor clinic flow, no or poor appointment systems, congested clinics with long waiting times, medication gaps, or medications not dispensed to patients after prescription (8, 9). These health facilities were supervised by the Uganda Ministry of Health (MoH) with ART funding from the Government of Uganda and donor agencies, including the Global Fund to Fight AIDS, Tuberculosis and Malaria and the U.S. President's Emergency Plan to Fight AIDS Relief (PEPFAR).

This was designed as an interventional cohort study, which tracked both experienced patients and those newly initiated on antiretroviral drugs (ARVs) using facility-based records. The major aim was to assess the impacts of the interventions on patients’ clinic attendance and adherence. Implemented over a period of 17 months in 2009 and 2010, the study consisted of collecting retrospectively pre-intervention data, an implementation phase of the interventions, and follow-up visits to the health facilities while prospectively collecting post-intervention data. During the pre-intervention period, we also visited each study facility to explain the purpose of the interventions to the clinic staff and to gain consensus on how to conduct the intervention.

The effects of the interventions were assessed by following two groups of patients using their clinical and dispensing records at the health facilities. In each facility, one randomly chosen cohort composed of experienced patients who had been on treatment at least 12 months prior to the interventions, and who were still in treatment 6 months before baseline. The other cohort, consecutively chosen, composed of all newly treated patients over their first 4 months, who initiated treatment during the 6-months prior to the baseline up to 4 months before the end of the follow-up period. After 4 months, these patients were transferred into the experienced cohort. The study was based on patients’ clinical records and facility dispensing records that were selected based on the criteria above. There was no direct involvement between the research team and the patients

At each health facility, we instituted two facility-level interventions. The impacts were measured at the patient level through time series analysis. These interventions aimed at improving patient flow and decongesting the clinics. We did not include a control group as the interventions implemented at the health facility level affected every patient attending the same facility.

### Description of the interventions

The first component of the interventions was to introduce an appointment system and fast-track the ‘refill-only’ patients. Patients categorized as ‘refill only’ were experienced patients, who had been on treatment for at least 12 months from baseline, with a self-reported record of 95% adherence to medication regimen. These were given appointments to come for refill of medications only and were fast-tracked through the clinic workflow without the need to be seen by clinicians or counselors if they did not report any complaints at triage. Traditionally, all patients would be required to pass through all stages of the clinic workflow, where they would be reviewed by the counselors and clinicians before being dispensed with refill medication. This was in spite of whether they had health complaints or not, and would subsequently lead to clinic congestions. The appointment system consisted of a book where the next review date would be recorded and patients were encouraged to return on that particular date. An appointment diary was also given to the patient to act as a reminder. Thus, the clinicians would be able to ascertain in advance which patients to expect on any particular day, and by the end of the clinic day, which patients had not honored their appointments. Currently in most Ugandan health facilities, patients are not given specific hours of appointment on the review dates. Furthermore, electronic dispensing is rare in the health facilities because of its associated technological challenges.

The second component of the interventions was to recommend that prescribers at the health facilities increase the numbers of days of medicines dispensed (longer prescription) from the traditional 30–60 days or 90 days for patients considered by the clinicians to be 95% adherent to their medication regimens. The aim was for such patients to make less frequent visits to the clinic, thus further reducing clinic congestion.

Health providers were provided with clear instructions to counsel the patients to report any problems during the scheduled visits or at any times without waiting for appointment days. This was to ensure that the patients remained in contact with clinicians at any time. In addition, the patients receiving longer prescription were to be seen by the clinician at least once every 6 months even when they had no complaints.

Apart from the above changes instituted by the research team, clinics continued to operate normally. The introduction of the appointment diaries and the appointment books provided the records of clinic attendance. The facilities also used these data for identification and follow-up of patients who missed appointments. Two weeks prior to implementing the interventions, we visited each study facility to explain the purpose of the interventions to the clinic staff and to gain consensus on how to conduct the intervention. After the intervention began, we made fortnightly supervisory visits, supplemented by telephone calls to ensure proper conduct throughout the post-intervention period. The research team was not directly involved in the practical implementation of the interventions.

### Sample selection

We aimed to follow a cohort of 100 experienced patients per facility, as recommended in WHO manuals on investigating longitudinal patterns of medication use (14, 15). Anticipating a 20% loss to follow-up during the study period, we thus set out to identify 125 experienced patients per facility. Assuming that a minimum of 80% of the total of 600 patients would complete all scheduled visits, this would mean a 95% confidence interval (CI) of ±4.5% on the sample-wide estimates of monthly rates, which was our primary focus. For newly treated patients, the sample size was determined by the rate of treatment initiation at each facility. We aimed to include all patients who were initiated on treatment during the study period.

### Data sources and data collection

Four research assistants used in this study were the same ones used in the earlier studies (8, 9). They were trained by the research team to retrieve prescription and clinical records from the patient files and the dispensing records, using methods recommended in the WHO manual on investigating longitudinal patterns of drug use (14, 15). They visited the health facilities with the intervention team to collect data but did not interact with patients. Data were collected from records in the clinic, pharmacy and appointment registers/diaries. During the 11 months of the post-intervention period, we collected data regarding the patients’ appointment dates, actual return dates, and the numbers of pills or days of medication dispensed. From these data, we computed the proportion of missed appointment and the days without medication (medication gaps) for each patient.

### Outcome measures

The following indicators were used to assess the impact of the interventions among the experienced cohort: the proportion of patients with any scheduled visits missed; the proportion of patients with more than 30 days of dispensed medication days; and the proportion of patients with 3 or more days without medication (medication gap).

A different analysis was performed for the newly treated cohort. This was because of the nature of enrolment of patients into this cohort – where some were enrolled during the post-intervention period. In this cohort, we investigated the event-rates of the patients experiencing a medication gap of seven or more days, and of 14 or more days during the first 120 days of treatment.

### Statistical analysis

We allowed 2 months after the initiation of the intervention before collecting post-intervention data. In this analysis, we have indicated this period as the ‘during’ period. The rationale was that the effects of the interventions would not accurately be measured immediately after instituting them. The interventions however continued to be implemented until the end of the study duration, overall 2 years.

The baseline characteristics of the two cohorts are presented using frequency tabulations for categorical variables, and descriptive summaries for the continuous variables. To assess the intervention effect, we first constructed graphical representations for each study outcome for the pre-, during and post-intervention periods. Overall aggregates of these outcome data per intervention periods have also been presented in tabular formats. We however excluded the ‘during’ intervention data from the modeling activities. For the experienced cohort, we modeled the intervention effect using mixed-effects models for binary responses with exchangeable correlation structure (16, 17). These models were adjusted for the patient-level demographic and clinical characteristics, including age, gender, initial treatment regimen, and pre-ART weight.

In the newly treated cohort, we used Cox proportional hazards survival models to determine the differences in event-rates of experiencing a medication gap of ≥7 days and ≥14 days after commencing therapy before and after the interventions. Patients were censored at the earliest of the following dates: on experiencing the outcome of interest; on the day of their last visit before reaching 120 days of treatment; or on the date of their last visit before the start of the intervention (for those initiating therapy within the pre-intervention period) or the end of follow-up (for those initiating therapy within the post-intervention period). We constructed the survival curves to depict the fraction experiencing the outcome for ease of interpretation. Statistical significance for the differences in the event-rates was assessed using the log-rank test. Cox proportional hazard models were fitted to determine the adjusted differences in survival between the intervention periods.

All analyses were performed using Stata/IC 10 (Stata Corp., College Station, TX, USA). All statistical inferences were assessed at the 5% type 1 error level, and all p-values presented were based on two-tailed tests.

### Ethical issues

Ethical clearance for the study was obtained from Makerere University Faculty of Medicine Research and Ethics Committee, while permission to conduct this study was obtained from the Uganda National Council of Science and Technology (UNCST). Consensus meetings were held with the heads of the health facilities before instituting the interventions in the health facilities. During these meetings, the practical implementation of the proposed interventions was discussed, given the workflow and operational settings of the health facilities would have to be adjusted. No written or verbal consent was sought from the patients, since the interventions were implemented by regular clinical staff at the facility from their routine records. No specific data were obtained directly from the patients during the study. The research team did not perform any data collection in the communities. Furthermore, all data were anonymized in all phases of data collection.

## Results

### Baseline characteristics of patients in the experienced cohort

In total, 720 patients with an average age of 38.8 years (SD±10.2 years) comprising 468 (65%) females and 252 (35%) males were recruited into the experienced cohort. Most patients (482/720, 67%) were aged 31–49, most had commenced therapy with advanced disease (540/720, 75% at WHO stages 3 or 4). The majority (662/720, 92%) commenced treatment with the stavudine (40)–lamivudine–nevirapine regimen (Table 1).

**Table 1:** Demographic and clinical characteristics of patients by cohort

Overall study population (N=1,481)	Experienced cohort^a^ (n=720)	Newly treated cohort^b^ (n=761)
Gender, n (%)		
Female	407 (65.2%)	315 (68.0%)
Male	217 (34.8%)	148 (32.0%)
Age in years, average (sd) n (%)	38.8 (10.2%)	35.82 (12.5)
18–30	113 (19.0%)	147 (32.5%)
31–49	400 (67.1%)	252 (55.8%)
50 and older	83 (14.0%)	53 (11.7%)
WHO stage at ART initiation, n (%)		
Stage 1	31 (5.5%)	32 (8.5%)
Stage 2	110 (19.5%)	120 (31.8%)
Stage 3	371 (66.0%)	193 (52.0%)
Stage 4	51 (9.1%)	32 (8.5%)
Initial ART regimen, n (%)		
AZT-3TC-EFV	5 (0.8%)	35 (7.5%)
AZT-3TC-NVP	35 (5.6%)	274 (58.8%)
TVD-EFV	2 (0.32%)	12 (2.6%)
TVD-NVP	1 (0.16%)	18 (3.9%)
d4T(30)-3TC-NVP	575 (91.9%)	114 (24.5%)
Others^c^	8 (1.3%)	13 (2.8%)
Average (sd) weight at ART initiation	53.35 (10.2)	51.35 (12.7)
Female	52.6 (10.3)	50.63 (11.5)
Male	54.0 (9.8)	52.81 (14.9)

a On ART at least 6 months prior to the index visit.

b Initiated ART within 6 months prior to the follow-up period.

c Other regimens include TDF-3TC-NVP, d4T (30)-3TC-EFV, and d4T (40)-3TC-NVP.

### Baseline characteristics of patients in the newly treated cohort

Overall, 761 patients (68% females) with an average age of 35.8 years (SD±12.5 years) were included in the newly treated cohort. More than half (60%) commenced therapy at WHO stages 3 or 4. The most common initial regimens were zidovudine–lamivudine–nevirapine (59%) and stavudine (30)–lamivudine–nevirapine (25%).

### Impact of the interventions in the experienced cohort

#### Any scheduled visits missed

**Fig. 1:**
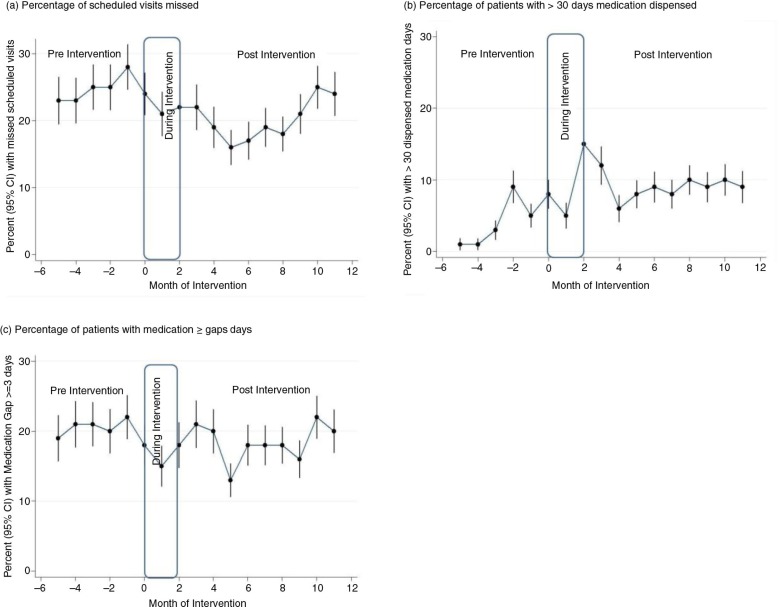
(a) Unadjusted monthly rates of attendance, (b) long duration dispensing, and (c) adherence in the experienced cohort.

The percentage of experienced patients who missed any scheduled appointments increased in the pre-intervention period, and then declined moderately during the post-intervention period (Fig. 1a). The decline was more pronounced during the first 6 months of the intervention before starting to rise again thereafter. The percentages of missed scheduled appointments in the pre- and post-intervention periods were 24.4 and 20.3%, respectively. Overall, the interventions were associated with a significant 33% reduction in the odds of missed visits (adjusted odds ratio (AOR): 0.67; 95% CI: 0.59–0.77) among experienced patients (Table 2).

**Table 2:** Changes in adherence-based indicators over time for experienced^a^ patients

	Unadjusted percent during period			Adjusted intervention effects	
		
Outcome	Pre-intervention (%)	During intervention (%)	Post intervention (%)	Odds ratio (95% CI)	p
Any scheduled visits missed	24.4	21.4	20.3	0.67 (0.59, 0.77)	<0.0001
Patients with >30 days dispensed medication days	4.3	8.0	9.3	2.35 (1.91, 2.89)	<0.0001
Patients with ≥3 days medication gaps	20.2	15.0	18.4	0.69 (0.60, 0.79)	<0.0001

a On ART at least 6 months prior to the index visit.

#### Patients with more than 30 days dispensed medication

After the interventions, there was an increase in the percentage of patients receiving medicines for longer periods at their scheduled refill (Fig. 1b). The percentage of visits where patients received more than 30 days of medication refills increased from 4.3% in the pre-intervention to 9.3% in the post-intervention period (Table 2). Overall, the interventions were associated with a significant 2.4 times increase in the odds of experienced patients receiving more than 30 days of medication refills (AOR: 2.35; 95% CI: 1.91–2.89).

#### Patients with medication gaps of 3 days or more

The percentage of experienced patients who were recorded to have medication gaps of 3 days or more at each visit declined over time (Fig. 1c). The percentages of visits with medication gaps were 20.2% in the pre-intervention, and 18.4% in the post-intervention period (Table 2). Overall, the interventions were associated with a significant 31% reduction in the odds of experiencing a medication gap of three or more days (AOR: 0.69; 95% CI: 0.60–0.79) among experienced patients.

### Impact of the interventions in the newly treated cohort

**Fig. 2:**
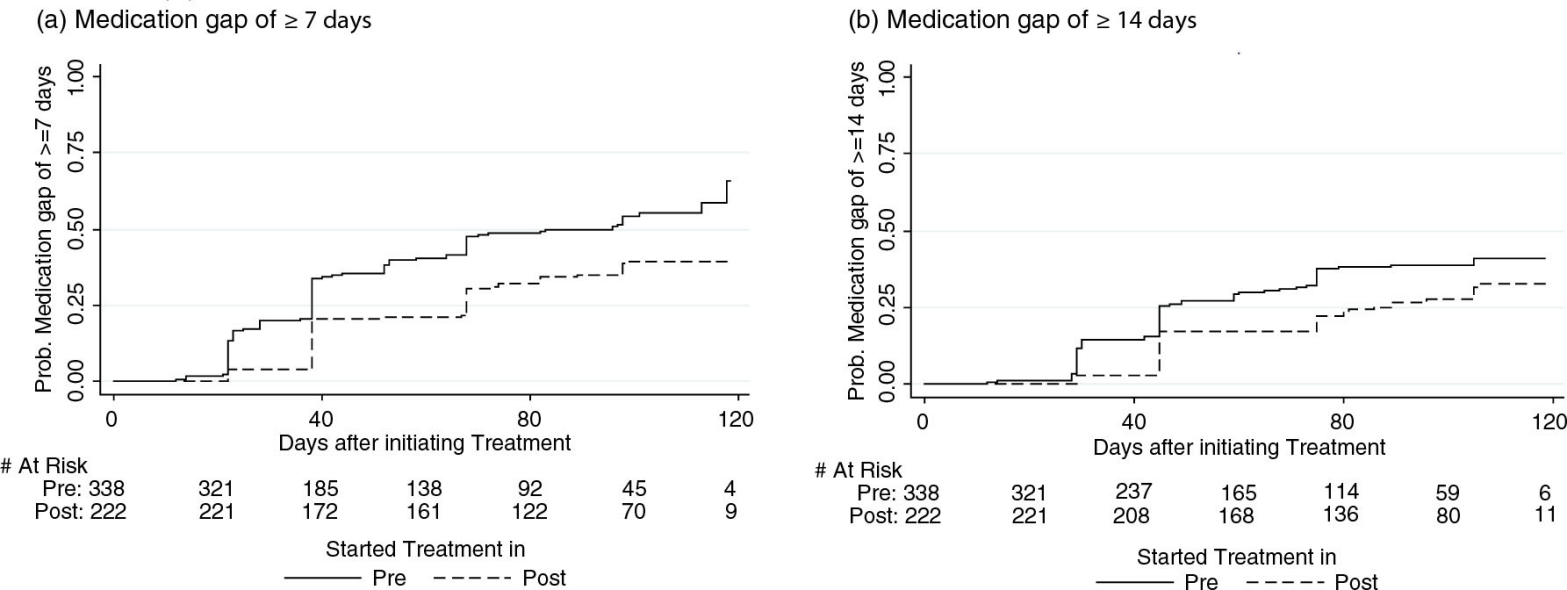
Probabilities of experiencing a medication gap of (a) ≥7 days or (b) ≥14 days during the first 120 days of antiretroviral treatment in the newly treated patient cohort.

Generally, the cumulative probability of newly treated patients experiencing long medication gaps was higher in the pre-intervention period as compared to the post-intervention period (Fig. 2a). The rates per 1,000 person-years of experiencing medication gaps of 7 or more days was 7.7 (95% CI: 6.5–9.0) in the pre-intervention period and 4.5 (95% CI: 3.6–5.6) in the post-intervention period (log-rank p-value <0.0001). Similarly, the rates per 1,000 person-years of experiencing medication gaps of 14 or more days was 4.8 (95% CI: 4.0 to 5.8) in the pre-intervention period and 3.1 (95% CI: 2.4–4.1) in the post-intervention period (log-rank p-value =0.0022). From the adjusted Cox proportional hazards models, the interventions were associated with a significant 44% reduction in the event-rates of experiencing medication gaps of 7 and more days (adjusted hazard rate (AHR): 0.56, 95% CI: 0.4–0.74), and a significant 38% reduction in the event-rates of experiencing medication gaps of 14 and more days (AHR: 0.62; 95% CI: 0.45–0.85) (Table 3).

**Table 3:** Probabilities of experiencing medication gaps of ≥7 days or ≥14 days during the first 120 days of treatment among newly treated patients

			Event rate per 1,000 person years		Adjusted proportional hazard estimates	
				
	Period	Number of events	Rate (95% CI) estimates	Log-rank p-value	Intervention effect (95% CI)	p-value
≥7 days	Pre	145	7.68 (6.52, 9.04)	<0.0001	[ref]	<0.0001
	Post	78	4.48 (3.58, 5.59)		0.56 (0.42, 0.74)	
≥14 days	Pre	102	4.81 (3.96, 5.83)	0.0022	[ref]	0.004
	Post	58	3.14 (2.43, 4.07)		0.62 (0.45, 0.85)	

## Discussion

Our findings show that clinical quality can be improved through a strategy whereby providers at facility level are encouraged to implement simple and feasible interventions to improve clinic patient flow that are adapted to the resources at their own facility, combined with minimal support supervision. We demonstrated a modest but significant 17% relative decrease in the percentage of patients who missed scheduled appointments. This translated into more patients keeping scheduled appointments, and thus improving adherence amongst such patients. Several studies have shown that appointment keeping is important for treatment success among patients on ART (18–20), and this study also demonstrated a decrease in the number of patients who missed appointments over time, although there was a tendency towards increase again during the last few months of follow-up.

One of the interventions was to encourage clinicians to increase the number of dispensed medication days for patients who had demonstrated their ability to remain consistently on therapy as a means to cut down the number of clinic visits and thereby decongest the clinic. We showed that the percentage of patients who were dispensed medication for more than 30 days more than doubled after the intervention. This practice has also been implemented elsewhere with considerable success (21–24). The greater than twofold increase in the proportion of patients who had thirty and more days of medication dispensed achieved in this study would help reduce crowds at the clinic and make the care process for these patients more convenient. However, dispensing greater quantities of medication may be limited by low stock levels or stock-outs in some resource-poor health facilities. Although we did not determine the stock levels of medicines at the facilities, this study has shown that increased medication days can be an effective way to decongest clinics and thereby improve adherence to ART even in health facilities with limited resources.

The percentage of experienced patients, who had medication gaps of three or more days, decreased significantly following the interventions. Among newly treated patients, the interventions significantly reduced the likelihood of experiencing a medication gap of seven or more days by about one-half, and the risk of a medication gap of 14 or more days by over one-third. The literature suggests that three or more days without medication may predict treatment failure among ART patients, while medication gaps of 14 or more days have been reported to be related to drug resistance development (25–27). The demonstrated reduction in the risk of experiencing medication gaps therefore shows that introducing a patient-appointment system, fast-tracking experience stable patients through the clinic, and dispensing larger prescriptions, makes the clinic less congested improving overall attendance and may be effectively executed as a way of improving patient adherence and guarding against treatment failure.

## Strengths and limitations

By engaging local health workers and empowering them to implement simple systems changes in their clinics, this study has shown that it is possible to improve patient attendance and adherence to ART without any increase in external resources. Introducing an appointment system, fast-tracking experienced patients, and dispensing larger prescriptions as part of routine service were intended to improve clinic flow, reduce congestion, and shortened clinic waiting time. These changes resulted in modest but significant improvements in adherence indicators. Other workers have shown that integrating interventions into routine service delivery does not only improve uptake of services but ensures sustainability (28).

The main strengths of the study are the design, the low-resource interventions and the robust analysis. The interventions were actively implemented by the staff at the respective facilities and accepted into their routine. They could be contextually adapted and institutionalized to promote sustainability. The relatively modest effects on attendance and adherence should be seen in light of the minimal input of extra resources and the huge benefits for those individuals, who maintain treatment. If the same type of interventions would be implemented at a national level with similar effects, another 15,000–20,000 of the 200,000 patients on treatment (29) would keep their appointments and thereby experience a better chance to stay adherent.

Despite the success of this approach, there are many challenges inherent in implementing real-world interventions in low-resource settings. Unfortunately, changes in policy and programs during the study period were critical events that may have affected the success of the interventions described in this study. For example, during the implementation period, the guidelines for first-line ARVs were changed. Many facilities were consequentially unable to consistently stock ARVs to support the new regimens resulting in an increased rate of stock-outs, and due to the retrospective nature of the study we were not able to collect data on daily stock availability. In addition, and most devastating, some ART support programs curtailed their activities when funding from PEPFAR was withdrawn (30), resulting in disruption of supply, thus directly influencing access to medicines and attendance. During the study, some facilities also introduced a nutrition program, which competed for resources and staff attention. However by reducing patient load using the recommended interventions, this may have made more time available for these other important tasks without the need for more resources. Given all of these external changes, it is remarkable that the simple measures resulted in meaningful and statistically significant improvements in attendance and adherence.

## Conclusions and recommendations

This study has demonstrated that easily implemented interventions designed to reduce clinic crowding, such as appointment systems, patient fast-tracking, and increasing medication days, and where existing resources are mobilized, are effective ways of improving adherence to ART among patients in Uganda. Key to the positive outcomes was the willingness of health workers to participate in the interventions to improve service quality. This way of implementing feasible interventions could be recommended for roll-out to all ART programs in Uganda and beyond. Success will largely depend on well-motivated staff members in the clinics, who are willing to make operational and workflow changes.

## References

[1] Agnarson AM, Masanja H, Ekstrom AM, Eriksen J, Tomson G, Thorson A (2010). Challenges to ART scale-up in a rural district in Tanzania: stigma and distrust among Tanzanian health care workers, people living with HIV and community members. Trop Med Int Health.

[2] Cooke GS, Tanser FC, Barnighausen TW, Newell ML (2010). Population uptake of antiretroviral treatment through primary care in rural South Africa. BMC Public Health.

[3] Hanefeld J (2010). The impact of Global Health Initiatives at national and sub-national level—a policy analysis of their role in implementation processes of antiretroviral treatment (ART) roll-out in Zambia and South Africa. AIDS Care.

[4] Uebel KE, Timmerman V, Ingle SM, van Rensburg DH, Mollentze WF (2010). Towards universal ARV access: achievements and challenges in Free State Province, South Africa. S Afr Med J.

[5] World Health Organization, UNAIDS (2006). Progress on global access to HIV antiretroviral therapy. A report on “3 by 5” and beyond. http://www.who.int/hiv/progreport2006_en.pdf.

[6] García de Olalla P, Knobel H, Carmona A, Guelar A, López-Colomés JL, Caylà JA (2002). Impact of adherence and highly active antiretroviral therapy on survival in HIV-infected patients. J Acquir Immune Defic Syndr.

[7] Bangsberg DR, Perry S, Charlebois ED, Clark RA, Roberston M, Zolopa AR (2001). Non-adherence to highly active antiretroviral therapy predicts progression to AIDS. AIDS.

[8] Chalker JC, Andualem T, Gitau LN, Ntaganira J, Obua C, Tadeg H (2010). Measuring adherence to antiretroviral treatment in resource-poor settings: the feasibility of collecting routine data for key indicators. BMC Health Serv Res.

[9] Gusdal AK, Obua C, Andualem T, Wahlstrom R, Tomson G, Peterson S (2009). Voices on adherence to ART in Ethiopia and Uganda: a matter of choice or simply not an option?. AIDS Care.

[10] Miller C, Tsoka MG (2012). ARVs and cash too: caring and supporting people living with HIV/AIDS with the Malawi Social Cash Transfer. Trop Med Int Health.

[11] Schouten EJ, Jahn A, Ben-Smith A, Makombe SD, Harries AD, Aboagye-Nyame F (2011). Antiretroviral drug supply challenges in the era of scaling up ART in Malawi. J Int AIDS Soc.

[12] Ross-Degnan D, Pierre-Jacques M, Zhang F, Tadeg H, Gitau L, Ntaganira J (2010). Measuring adherence to antiretroviral treatment in resource-poor settings: the clinical validity of key indicators. BMC Health Serv Res.

[13] Sanjobo N, Frich JC, Fretheim A (2008). Barriers and facilitators to patients’ adherence to antiretroviral treatment in Zambia: a qualitative study. SAHARA J.

[14] WHO and Management Sciences for Health (2011). How to investigate adherence to antiretroviral treatment: an indicator-based approach.

[15] WHO (1993). How to investigate drug use in health facilities: selected drug use indicators.

[16] Ng ESW, Carpenter JR, Goldstein H, Rasbash J (2006). Estimation in generalised linear mixed models with binary outcomes by simulated maximum likelihood. Stat Model.

[17] Rodriguez G, Goldman N (1995). An assessment of estimation procedures for multilevel models with binary responses. J Roy Stat Soc C Appl Stat.

[18] Dietz E, Clum GA, Chung SE, Leonard L, Murphy DA, Perez LV (2010). Adherence to scheduled appointments among HIV-infected female youth in five U.S. cities. J Adolesc Health.

[19] Farley J, Hines S, Musk A, Ferrus S, Tepper V (2003). Assessment of adherence to antiviral therapy in HIV-infected children using the Medication Event Monitoring System, pharmacy refill, provider assessment, caregiver self-report, and appointment keeping. J Acquir Immune Defic Syndr.

[20] Wagner GJ, Kanouse DE, Koegel P, Sullivan G (2004). Correlates of HIV antiretroviral adherence in persons with serious mental illness. AIDS Care.

[21] Gibson TB, Song X, Alemayehu B, Wang SS, Waddell JL, Bouchard JR (2010). Cost sharing, adherence, and health outcomes in patients with diabetes. Am J Manag Care.

[22] Patel UB, Ni Q, Clayton C, Lam P, Parks J (2010). An attempt to improve antipsychotic medication adherence by feedback of medication possession ratio scores to prescribers. Popul Health Manag.

[23] Toy EL, Beaulieu NU, McHale JM, Welland TR, Plauschinat CA, Swensen A (2011). Treatment of COPD: relationships between daily dosing frequency, adherence, resource use, and costs. Respir Med.

[24] Tuppin P, Neumann A, Danchin N, de Peretti C, Weill A, Ricordeau P (2010). Evidence-based pharmacotherapy after myocardial infarction in France: adherence-associated factors and relationship with 30-month mortality and rehospitalization. Arch Cardiovasc Dis.

[25] Bangsberg DR, Moss AR, Deeks SG (2004). Paradoxes of adherence and drug resistance to HIV antiretroviral therapy. J Antimicrob Chemother.

[26] Parienti JJ, Das-Douglas M, Massari V, Guzman D, Deeks SG, Verdon R (2008). Not all missed doses are the same: sustained NNRTI treatment interruptions predict HIV rebound at low-to-moderate adherence levels. PLoS One.

[27] Bangsberg DR, Kroetz DL, Deeks SG (2007). Adherence–resistance relationships to combination HIV antiretroviral therapy. Curr HIV/AIDS Rep.

[28] Kasenga F, Byass P, Emmelin M, Hurtig A-K (2009). The implications of policy changes on the uptake of a PMTCT programme in rural Malawi: first three years of experience. Glob Health Action.

[29] AVERT HIV and AIDS in Uganda. http://www.avert.org/aids-uganda.htm.

[30] Mugyenyi P (2009). Flat-line funding for PEPFAR: a recipe for chaos. Lancet.

